# The cingulate cortex and limbic systems for emotion, action, and memory

**DOI:** 10.1007/s00429-019-01945-2

**Published:** 2019-08-26

**Authors:** Edmund T. Rolls

**Affiliations:** 1 Oxford Centre for Computational Neuroscience, Oxford, UK; 2 Department of Computer Science, University of Warwick, Coventry, CV4 7AL UK

**Keywords:** Cingulate cortex, Limbic systems, Hippocampus, Orbitofrontal cortex, Emotion, Memory, Depression

## Abstract

Evidence is provided for a new conceptualization of the connectivity and functions of the cingulate cortex in emotion, action, and memory. The anterior cingulate cortex receives information from the orbitofrontal cortex about reward and non-reward outcomes. The posterior cingulate cortex receives spatial and action-related information from parietal cortical areas. It is argued that these inputs allow the cingulate cortex to perform action–outcome learning, with outputs from the midcingulate motor area to premotor areas. In addition, because the anterior cingulate cortex connects rewards to actions, it is involved in emotion; and because the posterior cingulate cortex has outputs to the hippocampal system, it is involved in memory. These apparently multiple different functions of the cingulate cortex are related to the place of this proisocortical limbic region in brain connectivity.

**Electronic supplementary material:**

The online version of this article (10.1007/s00429-019-01945-2) contains supplementary material, which is available to authorized users.

## Introduction: the cingulate cortex and other limbic structures

A key area included by Broca in his limbic lobe (Broca 1878) is the cingulate cortex, which hooks around the corpus callosum. The term limbic used by Broca referred to structures that are at the border or edge (the literal meaning of limbic) of the hemispheres (when seen in medial view), and led to the development of the concept of a limbic system (Pessoa and Hof 2015). Other limbic structures include the hippocampus, and the amygdala (which has major connections with the orbitofrontal cortex). These structures appear to have very different connections and functions. The amygdala and orbitofrontal cortex are key structures involved in emotion and reward value with connections from ventral stream processing areas that decode ‘what’ the stimulus is (Rolls 2014b, 2016a, 2019a, b). The hippocampus is a key structure in episodic memory with inputs from the dorsal stream cortical areas about space, action, and ‘where’ events occur, as well as from the ‘what’ ventral processing stream (Kesner and Rolls 2015; Rolls 2018b). Because of the different connectivity and functions of these limbic structures (amygdala, orbitofrontal cortex, and hippocampus) in emotion and in memory, it has been suggested that the concept of a single ‘limbic system’ is not realistic, and that we should consider separately the connectivity and functions of different limbic structures in emotion and memory (Rolls 2015).

However, that leaves the cingulate cortex in an interesting position straddling the emotional and memory domains. The anterior cingulate cortex receives inputs from the orbitofrontal cortex and amygdala which receive from ventral stream areas. The posterior cingulate cortex receives from dorsal stream areas including the parietal cortex and has connections to the hippocampal memory system. Moreover, there is evidence relating the cingulate cortex to what is apparently something else, action–outcome learning, in which actions are learned to obtain goals based on the outcomes, the rewards and punishers, received for different actions (Rushworth et al. 2012; Kolling et al. 2016; Rolls 2019a).

In this paper, I provide a framework for understanding the connectivity and functions of the cingulate cortex, and how the cingulate cortex can be involved in at least three important functions, emotion, action–outcome learning, and memory. I consider the evidence on the connectivity and functions of the different parts of the cingulate cortex, and then in the synthesis I produce an integrated conceptualization of how the cingulate cortex, with its proisocortical structure and type of connectivity, fits in to our overall conceptual understanding of the functions performed by different cortical areas, and by other limbic structures (Rolls 2016a). Proisocortical areas can be understood in terms of brain structure and function as forming connectional bridges between neocortical areas and areas such as the hippocampus (which is termed allocortex) (Pandya et al. 2015) (see “Connectivity of the cingulate cortex with the hippocampal memory system”). This is a conceptual review of the structure and functions of the cingulate cortex, and introduces new concepts on how it relates to other limbic and cortical structures, rather than an exhaustive review. The cytoarchitectural details of the human cingulate cortex are provided in Fig. S1 (Vogt 2009, 2016).

Many previous contributions have been valuable in leading towards an understanding of the cingulate cortex (Devinsky et al. 1995; Vogt 2009, 2016; Rushworth et al. 2011). This paper focusses on research on the cingulate and orbitofrontal cortex in primates including humans, rather than on research in rodents (Izquierdo 2017; Wikenheiser and Schoenbaum 2016), because of the great development of these regions in primates (Passingham and Wise 2012; Rolls 2014a, 2018a, 2019a). For example, rodents have no posterior cingulate cortex (Vogt 2009) and most of the orbito-frontal cortex (apart from the agranular areas posteriorly) may not be present in rodents (Passingham and Wise 2012; Rolls 2019a).

**Fig. 1:**
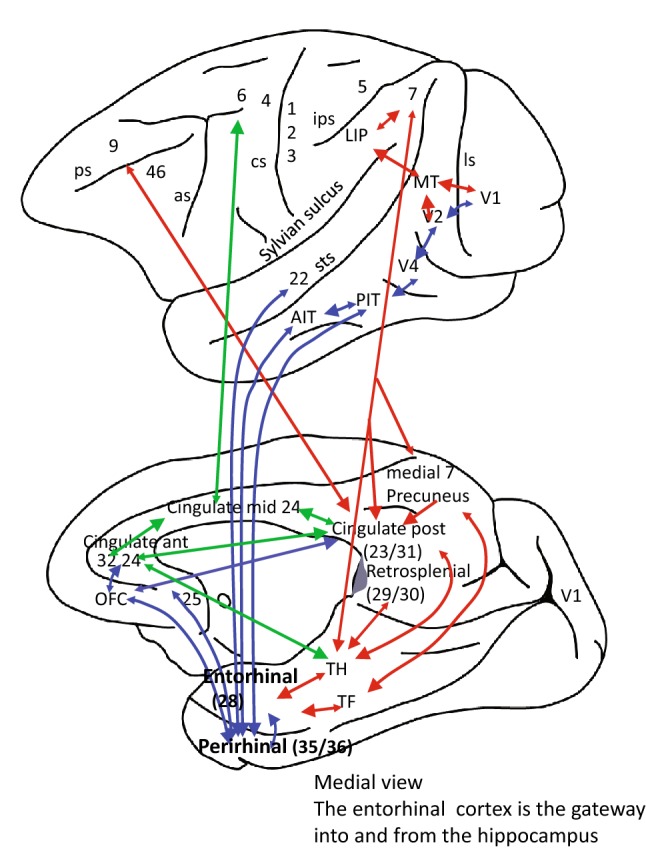
The connections of the anterior and posterior cingulate cortex with their input areas, and their outputs to the hippocampal memory system. A medial view of the macaque brain is shown below, and a lateral view is above. The green arrows show the convergence of reward or outcome information from the ACC and of information about actions from the posterior cingulate cortex to the midcingulate motor area, which then projects to premotor areas including the premotor cortex area 6 and the supplementary motor area. This provides connectivity for action–outcome learning. The anterior cingulate cortex receives reward outcome information from the orbitofrontal cortex (OFC). The posterior cingulate cortex (23 and 31) receives information about actions from the parietal cortex. This cingulate connectivity is compared with that of the hippocampus, which receives information from the ventral ‘what’ processing stream (blue) and the dorsal ‘where’ or ‘action’ processing stream (red), as described in the text. as, arcuate sulcus; cs, central sulcus; ips, intraparietal sulcus; ios, inferior occipital sulcus; ls, lunate sulcus; retrosplenial cortex (29, 30) is the small region in primates including humans behind the splenium of the corpus callosum shaded grey: it is present in rodents, which do not have a posterior cingulate cortex (Vogt 2009); sts, superior temporal sulcus; 4, 6, motor and premotor cortex. Developed from Rolls and Wirth (2018)

**Fig. 2:**
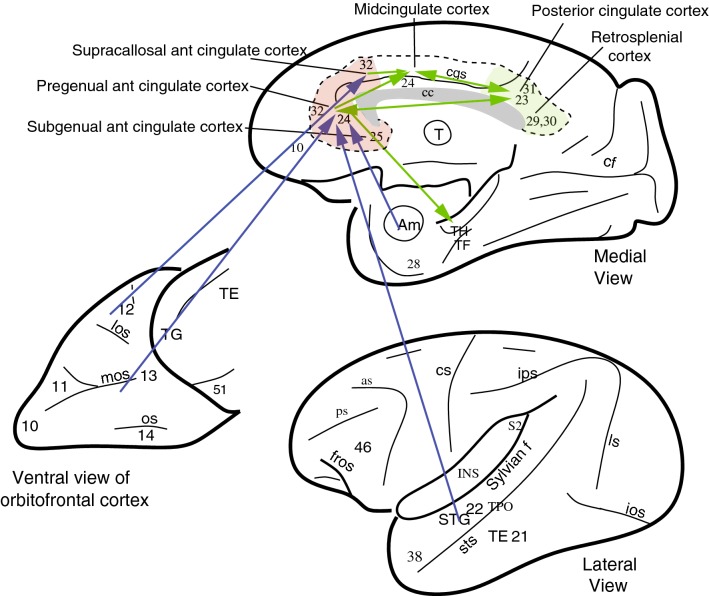
Connections of the anterior cingulate cortex shown on views of the primate brain (see text). The arrows show the main direction of connectivity, but there are connections in both directions. The supracallosal anterior cingulate cortex is also termed the anterior part of the midcingulate cortex, and is distinct from the posterior part of the midcingulate cortex (pMidcingulate). Connections reach the pregenual cingulate cortex especially from the medial/mid-orbitofrontal cortex; and reach the supracallosal anterior cingulate cortex especially from the lateral orbitofrontal cortex. Connections to the anterior cingulate cortex from the temporal lobe are from the (auditory) superior temporal gyrus (STG), from the visual and auditory cortex in the superior temporal sulcus; and from the amygdala. as, arcuate sulcus; cc, corpus callosum; cf., calcarine fissure; cgs, cingulate sulcus; cs, central sulcus; ls, lunate sulcus; ios, inferior occipital sulcus; mos, medial orbital sulcus; os, orbital sulcus; ps, principal sulcus; sts, superior temporal sulcus; Sf, Sylvian (or lateral) fissure (which has been opened to reveal the insula); Am, amygdala; INS, insula; TE (21), inferior temporal visual cortex; STG (22), superior temporal gyrus auditory association cortex; TF and TH, parahippocampal cortex; TPO, multimodal cortical area in the superior temporal sulcus; 38, TG, temporal pole cortex; 13, 11, medial orbitofrontal cortex; 12, lateral orbitofrontal cortex; 23, 31 posterior cingulate cortex; 29, 31 retrosplenial cortex; 51, olfactory (prepyriform and periamygdaloid) cortex. A cytoarchitectural map of the human cingulate cortex is provided in Fig. S1

An overview of the concepts about the cingulate cortex considered here follows, to provide a framework for understanding the evidence included in this paper. Some of the connections and regions of the cingulate cortex are shown in Figs. 1 and 2.

In humans, the pregenual anterior cingulate cortex receives information related to reward from the medial orbitofrontal cortex, and related to punishment and not receiving reward from the lateral orbitofrontal cortex. These orbitofrontal cortex areas represent information about value, not about actions or responses, as shown by neuronal recordings in macaques. The value representation received by the anterior cingulate cortex includes information about the outcome of actions, that is, whether reward or punishment has been received, and is used by the cingulate cortex for learning the action to perform to obtain a reward or avoid a punisher. This is termed ‘action–outcome learning’. The anterior cingulate cortex is implicated in emotion, because it is involved in linking reward and punishment information, which elicit emotional responses, to behaviour, and, in particular, to actions. The subgenual cingulate cortex (area 25) may link rewards and punishers to autonomic output. The posterior cingulate cortex receives information about actions from the parietal cortex, including areas 7a, VIP and LIP laterally, and area 7m medially (as well as some inputs from ventral stream temporal lobe areas) (Vogt and Laureys 2009). The posterior cingulate cortex is therefore implicated in spatial including visuospatial processing. A concept is that the posterior cingulate action-related information is brought together with the anterior cingulate cortex outcome-related information, and via the midcingulate cortex the result of action–outcome learning can influence premotor areas that receive information from the midcingulate cortex (Fig. 1). The posterior cingulate cortex in addition has major connectivity with parahippocampal areas TF and TH, which in turn project spatial information to the entorhinal cortex and thereby into the hippocampal episodic memory system. The posterior cingulate cortex provides a route for spatial including visuospatial information to reach the hippocampus, where it can be combined with object and reward-related information to form episodic memories (Rolls 2016a, 2018b; Rolls and Wirth 2018). The posterior cingulate cortex is thereby also implicated in memory.

These concepts show how different parts of the cingulate cortex can be involved in reward and punishment processing and thereby in emotion; in learning actions to perform to obtain rewards and avoid punishers and thereby in action; and in memory. Because of these different functions and connectivities, the cingulate cortex cannot easily be considered as part of a single limbic system, but instead contributes to some of the different functions performed by different limbic structures that are related to their connections to different neocortical areas (Rolls 2015).

## The anterior cingulate cortex

### Conceptual framework

The anterior cingulate cortex (ACC), itself a limbic structure, has connections with a set of other limbic and related areas including the amygdala and orbitofrontal cortex (OFC) involved in emotion and reward-related processing (see Fig. 1, and for details “Anterior cingulate cortex: anatomy and connections”) (Rolls 2014a, 2018a). This set of limbic and related structures related to the ventral or ‘what’ processing streams (Ungerleider and Haxby 1994), and these ventral processing streams themselves, provide a major source of ‘what’ and ‘reward’ input into the hippocampal memory system via the perirhinal and entorhinal cortex (blue in Fig. 1) (Rolls 2018b; Rolls and Wirth 2018).

The posterior cingulate cortex has connections from parietal structures such as the precuneus and lateral parietal areas and is involved in spatio-topographical and related memory functions (Cavanna and Trimble 2006; Rolls 2015; Rolls and Wirth 2018; Leech and Sharp 2014; Kircher et al. 2002; Vogt 2009; Vogt and Pandya 1987; Vogt and Laureys 2009). This limbic region related to the dorsal or ‘where’ processing systems (Ungerleider and Haxby 1994) provides a second major source of input into the hippocampal memory system, via the parahippocampal gyrus (areas TF and TH) and the entorhinal cortex (red in Fig. 1) (Rolls 2018b; Rolls and Wirth 2018). Because the ACC and its related limbic areas, and the posterior cingulate cortex and its related areas, have such different connections and functions, it has been argued that we should no longer think of a single limbic system, but instead of two (or more) limbic processing systems (Rolls 2015).

However, a key concept is that the orbitofrontal/anterior cingulate/amygdala set of limbic areas related to ventral steam processing, and the posterior cingulate cortex related to dorsal stream processing, enable the ventral and dorsal processing streams to be brought together in the hippocampus, so that we can form memories of ‘what’ happened ‘where’, which is prototypical of episodic memory (Rolls 2016a, 2018b; Kesner and Rolls 2015). In an interesting twist, there is in fact a connection from the orbitofrontal cortex to the posterior cingulate cortex (Vogt and Laureys 2009), which provides a path for reward- and punishment-related information to enter the hippocampus via the dorsal route as well as by the ventral route (Rolls 2018b; Rolls and Wirth 2018).

This conceptual framework is developed a little more next, with the more detailed evidence provided later. It should be noted that this framework applies to primates including humans, with the principles of operation being considerably different in rodents, due to the much less well-developed orbitofrontal cortex, and visual and even taste cortical processing areas (Rolls 2016a, 2018a, 2019a).

The orbitofrontal cortex represents the reward value of stimuli (Rolls 2000, 2014a, 2018a, 2019a, b; Tremblay and Schultz 1999; Rolls et al. 1989; Small et al. 2001). It is in a sense an output region for all the sensory systems, including taste, olfaction, visual, auditory, and somatosensory, that represents ‘what’ a stimulus is, and uses that information to build what are frequently multimodal representations but in value space rather than in ‘what’ or stimulus identity space. Orbitofrontal cortex neurons focus on reward value representations for stimuli and know little about actions.

The orbitofrontal cortex sends inputs to the ACC about the value of stimuli, that is, about goals including the value of outcomes (the reward received) and the expected value. The ACC in combination with the midcingulate motor area, which contains representations of actions, interfaces actions to outcomes (rewards or punishers received) using action–outcome learning, and also takes into account the cost of actions to obtain the goal when selecting actions (Rushworth et al. 2012; Kolling et al. 2016; Rolls 2019a). The anterior and midcingulate cortical areas are thus relevant to emotion, for they implement the instrumental goal-directed actions that the instrumental reinforcers involved in emotion produce (Rolls 2014a, 2019a, b). In the context of its representations of value, damage to the anterior cingulate areas does influence emotion (Rolls 2014a, 2019a).

The ACC operates as a system that performs goal-directed actions to obtain rewards and avoid punishers, and takes into account the outcomes received after actions, in that it is sensitive to devaluation of the goal, and will not select an action if the goal has been devalued. This is in contrast to the basal ganglia, which implement a stimulus–motor response mapping which becomes automated as a habit after much learning, and is not sensitive to devaluation of the goal (Rolls 2014a, 2019a, b).

### Anterior cingulate cortex: anatomy and connections

The ACC areas occupy approximately the anterior third of the cingulate cortex (Fig. 2). They are separate from a midcingulate/cingulate motor area (Vogt 2009; Vogt et al. 2003) that may be involved in action selection (Rushworth et al. 2004, 2011; Noonan et al. 2011). The ACC includes area 32, the pregenual cingulate cortex (where genu refers to the knee of the corpus callosum); area 25, the subgenual cingulate cortex; and a part of area 24 (Fig. 2) (Price 2006a, b; Öngür et al. 2003; Ongür and Price 2000). [It is however noted that the midcingulate cortex has been described as having an anterior and a posterior part, with the criteria including cytoarchitecture (Vogt 2016) (see Fig. S1), and that the anterior part may overlap with or be similar to what is described as the supracallosal part of the ACC in “Anterior cingulate cortex: functional neuroimaging and neuronal activity”.]

As shown in Fig. 2, the ACC receives strong inputs from the orbitofrontal cortex (Carmichael and Price 1995; Morecraft and Tanji 2009; Vogt 2009; Carmichael and Price 1996; Devinsky et al. 1995; Vogt and Pandya 1987; Vogt 2019). The ACC is also characterized by connections with the amygdala (Carmichael and Price 1995; Morecraft and Tanji 2009; Vogt 2009). The ACC also has connections with some temporal cortical areas involved in memory including the parahippocampal gyrus (which provides via the entorhinal cortex a bridge to the hippocampus); and with the rostral superior temporal gyrus, the auditory superior temporal gyrus, and the dorsal bank of the superior temporal sulcus (Vogt 2009; Rolls 2016a, 2018b; Saleem et al. 2008; Insausti et al. 1987) (see Fig. 2). [The cortex in the superior temporal sulcus contains visual neurons that respond to face expression, gesture, and head movement (Hasselmo et al. 1989a, b).]

In more detail, a ‘medial prefrontal network’ (mainly ACC) selectively involves medial areas 14r, 14c, 24, 25, 32, and 10m, rostral orbital areas 10o and llm, and agranular insular area Iai in the posterior orbital cortex in macaques (Carmichael and Price 1996). An ‘orbital’ prefrontal network links most of the areas within the orbital cortex, including areas Iam, Iapm, Ial, 121, 12m, and 12r in the caudal and lateral parts of the orbital cortex, with areas 131, 13m, and 13b in the central orbital cortex, which have further onward connections to the rostral orbital area 11l (Carmichael and Price 1996; Price 2006b; Ongür and Price 2000). Several orbital areas (including 13a, 12o, and 11m) have connections with both the medial and orbital networks. Many of these areas are shown in Fig. 2. It is very interesting that this medial prefrontal network has connections with the posterior cingulate/retrosplenial cortex and parahippocampal cortex (Saleem et al. 2008), and has access to the hippocampus in this way, whereas the orbitofrontal cortex has projections to the perirhinal cortex (Saleem et al. 2008), and thus has access to the hippocampus via a more ventral route (Fig. 1).

**Fig. 3:**
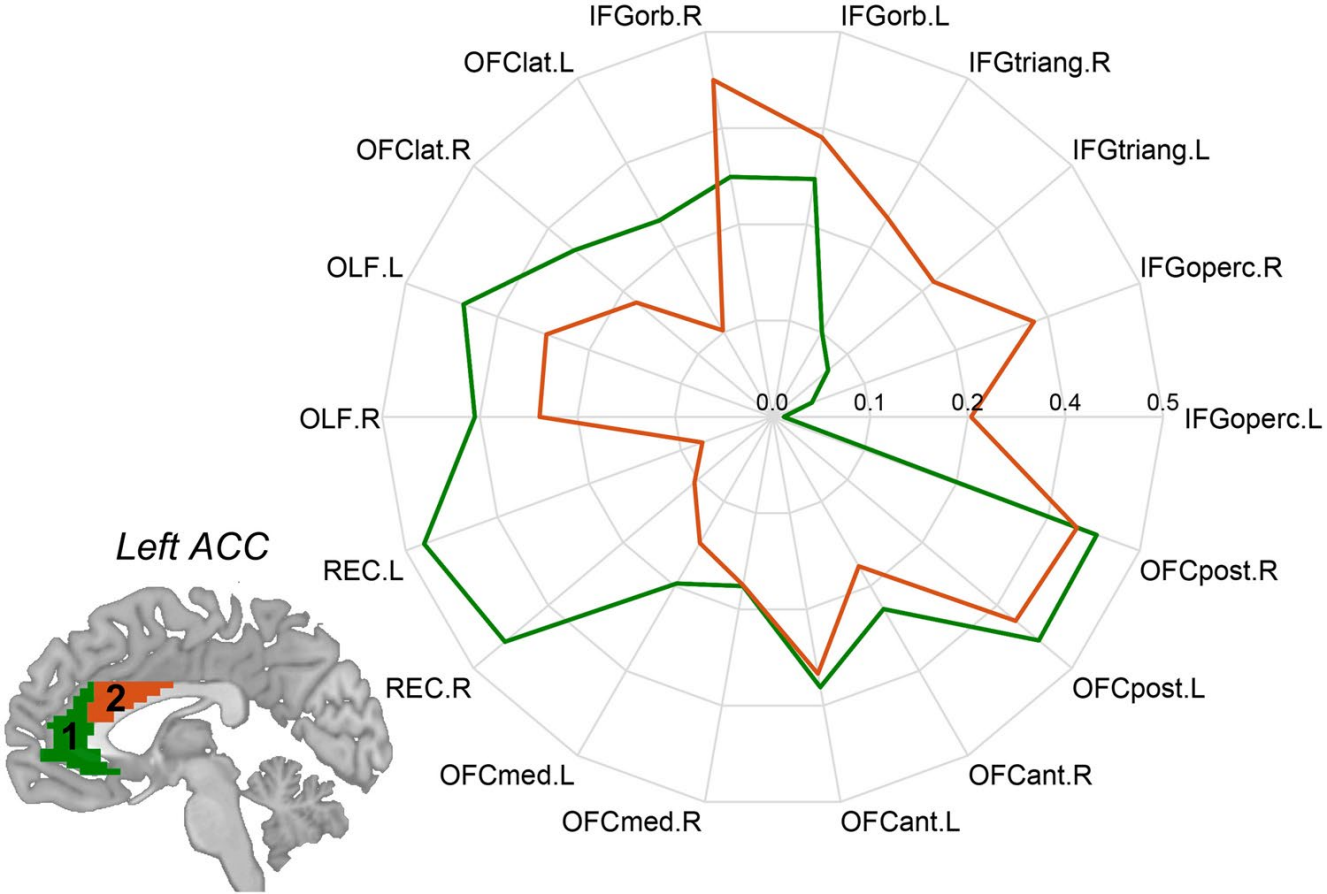
Voxel-level parcellation of the left anterior cingulate cortex (ACC) based on its functional connectivity in healthy individuals with other brain areas. The correlations (*r*) are the distance from the centre of the circular plot. The pregenual and subcallosal subdivision (1, green) has strong functional connectivity with the medial orbitofrontal cortex and connected areas (AAL2 areas from OLF to OFCpost). The supracallosal subdivision (2, red) has strong functional connectivity with the lateral orbitofrontal cortex area IFGorb and with adjacent inferior frontal gyrus areas (IFGtriang to IFGoperc). The parcellation was similar on the right. The AAL2 is the automated anatomical labelling atlas, which shows the abbreviations used (Rolls et al. 2015)

**Fig. 4:**
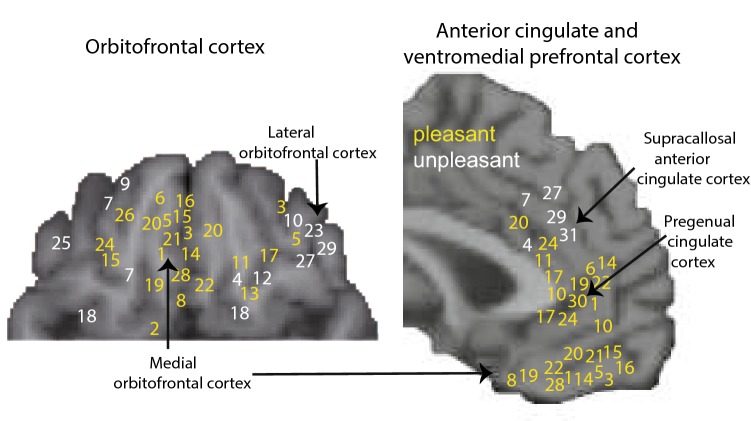
Maps of subjective pleasure in the orbitofrontal cortex (ventral view) and anterior cingulate cortex (sagittal view). Yellow: sites where activations correlate with subjective pleasantness. White: sites where activations correlate with subjective unpleasantness. The numbers refer to effects found in specific studies. Taste: 1, 2; odour: 3–10; flavour: 11–16; oral texture: 17, 18; chocolate: 19; water: 20; wine: 21; oral temperature: 22, 23; somatosensory temperature: 24, 25; the sight of touch: 26, 27; facial attractiveness: 28, 29; erotic pictures: 30; laser-induced pain: 31. [After Grabenhorst and Rolls (2011) who provide references to the original studies.]

A very interesting new finding about ACC connectivity in relation to what follows is that the medial orbitofrontal cortex has strong functional connectivity with the pregenual cingulate cortex, in both of which rewards are represented; and that the lateral orbitofrontal cortex (and inferior frontal gyrus) has strong functional connectivity with the supracallosal, more dorsal, ACC area, both of which are activated by unpleasant aversive stimuli (Rolls et al. 2018). This was shown in a resting state fMRI investigation with 254 healthy participants (Rolls et al. 2018). [Functional connectivity refers to correlations between the fMRI BOLD signal in different brain regions, and may include trans-synaptic effects as well as reflecting direct connections (Van Essen et al. 2019). The concept is that brain areas with high correlations in their signals are likely to have direct or indirect connections. I aim to include the term ‘functional’ when this type of connectivity is referred to.] Parcellation (to identify different clusters of voxels with similar functional connectivity) was performed based on the functional connectivity of individual ACC voxels in the control participants (Fig. 3). (The functional connectivity was measured by the correlation of the resting state fMRI signals between pairs of voxels.) A pregenual and subcallosal subdivision (1, green) has strong functional connectivity with the medial orbitofrontal cortex and connected areas (Fig. 3), which are implicated in reward (Fig. 4). The supracallosal subdivision (2, red), which is activated by unpleasant stimuli and non-reward, has high functional connectivity with the lateral orbitofrontal cortex and adjacent inferior frontal gyrus areas (Fig. 3), also activated by unpleasant stimuli (Fig. 4) (Rolls et al. 2018). These functional connectivities provide support for the hypothesis that the reward-related medial orbitofrontal cortex provides inputs to the pregenual cingulate cortex, also activated by rewards; and that the lateral orbitofrontal cortex, implicated in effects of non-reward and punishers, provides inputs to the supracallosal part of the ACC, which is also activated by unpleasant stimuli (Rolls et al. 2018; Rolls 2019a).

It should be noted that the medial prefrontal network includes area 14 (gyrus rectus) in the most medial part of the orbital cortex, and that area 14 should not be included in Price and colleagues’ ‘orbital prefrontal network’ (Carmichael and Price 1996). Their ‘orbital prefrontal network’ includes areas on the posterior, central and lateral, orbital surface (agranular insular areas Ial, Iam, Iapl and Iapm, and orbital areas 13b, 13l, 13m, 11l, 12r, 12m and 12l, see Fig. 2), which is described as the orbitofrontal cortex (Rolls 2014a, 2018a, 2019a, b). The term ‘ventromedial prefrontal cortex’ (vmPFC) is not well defined anatomically, and refers generally to a medial and ventral region of the prefrontal cortex which probably can be taken to include the ventral parts of the ‘medial prefrontal network’ of Price and colleagues, and also probably medial parts of the orbitofrontal cortex and medial area 10 (Rolls 2019a). Indeed, different parts of the ventromedial prefrontal cortex have different functional connectivity with other brain areas (Rolls et al. 2019a). The ventromedial prefrontal cortex may play a particular role in decision-making, especially in the choice between different rewards (Grabenhorst and Rolls 2011; Rolls et al. 2010a, b; Glascher et al. 2012; Rolls 2019a).

The outputs of the ACC reach further back in the cingulate cortex towards the midcingulate cortex, which includes the cingulate motor area (Vogt 2009; Vogt et al. 1996; Morecraft and Tanji 2009; Vogt 2016). The ACC also projects forward to the medial prefrontal cortex area 10 (Price 2006b; Ongür and Price 2000). Another route for output is via the projections to the striatum/basal ganglia system. The connections with the temporal lobe have been described above (Saleem et al. 2008). The ACC, including the subgenual cingulate cortex area 25, has outputs that can influence autonomic/visceral function via the hypothalamus, midbrain periaqueductal grey, and insula, as does the orbitofrontal cortex (Rempel-Clower and Barbas 1998; Critchley and Harrison 2013; Price 2006b; Ongür and Price 2000).

### Anterior cingulate cortex: functional neuroimaging and neuronal activity

#### A framework

The pregenual and the adjoining dorsal supracallosal anterior cingulate areas (Fig. 2) can be thought of as areas that allow information about rewards and outcomes received from the orbitofrontal cortex to be linked, via the cingulum fibre bundle, to action-related information in the midcingulate cortex (Fig. 1). The orbitofrontal cortex represents value, but not actions or behavioural responses (Thorpe et al. 1983; Rolls 2019a, b; Padoa-Schioppa and Assad 2006; Grattan and Glimcher 2014), and therefore projects value-related information to the ACC where the value-related information can be used to guide actions. Combining in the cingulate cortex information about particular rewards received for particular actions, and the particular costs involved, is essential for associating actions with the value of their outcomes, to select an action that will lead to the desired goal (Walton et al. 2003; Rushworth et al. 2007, 2011; Grabenhorst and Rolls 2011; Rolls 2014a; Kolling et al. 2016). Indeed, consistent with its strong connections to motor areas (Morecraft and Tanji 2009), lesions of the ACC impair reward-guided action selection (Rudebeck et al. 2008; Kennerley et al. 2006); in humans, the ACC is activated when information about outcomes guides choices (Walton et al. 2004), and neurons in the ACC encode information about actions, outcomes, and prediction errors for actions (Matsumoto et al. 2007; Luk and Wallis 2009; Kolling et al. 2016). For example, if information about three possible outcomes (different juice rewards) had to be associated with two different actions, information about both specific actions and specific outcomes was encoded by neurons in the ACC (Luk and Wallis 2009).

#### Pregenual anterior cingulate representations of reward value, and supracallosal anterior cingulate representations of punishers and non-reward

Functional magnetic resonance neuroimaging (fMRI) studies provide evidence for somewhat separate representations of rewarding, positively affective (pleasant) stimuli in the pregenual cingulate cortex (yellow in Fig. 4), and of negative, unpleasant, stimuli just posterior to this above the corpus callosum in the ACC (white in Fig. 4) (Rolls 2009; Grabenhorst and Rolls 2011). Pain activates an area typically 10–30 mm posterior to and above the most anterior (i.e. pregenual) part of the ACC (Fig. 4) (Rolls et al. 2003b; Vogt et al. 1996; Vogt and Sikes 2000). Pleasant touch activated the pregenual cingulate cortex (Fig. 4) (Rolls et al. 2003b; McCabe et al. 2008). Pleasant temperature applied to the hand also produces a linear activation proportional to its subjective pleasantness in the pregenual cingulate cortex (Rolls et al. 2008b). Somatosensory oral stimuli including viscosity and the pleasantness of the texture of fat in the mouth also activate the pregenual cingulate cortex (de Araujo and Rolls 2004; Grabenhorst et al. 2010b). Pleasant (sweet) taste also activates the pregenual cingulate cortex (de Araujo et al. 2003a; de Araujo and Rolls 2004) where attention to pleasantness (Grabenhorst and Rolls 2008) and cognition (Grabenhorst et al. 2008a) also enhances activations. Pleasant odours also activate the pregenual cingulate cortex (Rolls et al. 2003a), and these activations are modulated by word-level top-down cognitive inputs that influence the pleasantness of odours (de Araujo et al. 2005), and also by top-down inputs that produce selective attention to odour pleasantness (Rolls et al. 2008a). Unpleasant odours activate further back in the ACC (Rolls et al. 2003a). The pregenual cingulate cortex is also activated by the ‘taste’ of water when it is rewarding because of thirst (de Araujo et al. 2003b), by the flavour of food (Kringelbach et al. 2003), and by monetary reward (O’Doherty et al. 2001). Moreover, the outcome value and the expected value of monetary reward activate the pregenual cingulate cortex (Rolls et al. 2008c). Figure 4 shows the sites of some of these activations.

In these studies, the anterior cingulate activations were linearly related to the subjective pleasantness or unpleasantness of the stimuli, providing evidence that the ACC represents value on a continuous scale (Fig. 4), which is characteristic of what is found in the sending region, the orbitofrontal cortex (Rolls 2019a, b). Moreover, evidence was found that there is a common scale of value in the pregenual cingulate cortex, with the affective pleasantness of taste stimuli and of thermal stimuli delivered to the hand producing identically scaled BOLD activations (Grabenhorst et al. 2010a). The implication is that the ACC contains a value representation used in decision-making, but that the decision itself may be made elsewhere. Decisions about actions that reflect the outcomes represented in the ACC may be made further posterior towards the midcingulate cortex. Decisions about the value of stimuli may be made in the medial prefrontal cortex area 10 (or ventromedial prefrontal cortex, VMPFC) as shown by fMRI evidence (Grabenhorst et al. 2008b; Rolls and Grabenhorst 2008; Rolls et al. 2010a, b). Consistent with this, in macaques single neurons in the ventromedial prefrontal cortex rapidly come to signal the value of the chosen offer, suggesting the circuit serves to produce a choice (Strait et al. 2014), consistent with the attractor model of decision-making (Rolls et al. 2010a, b; Rolls 2014a, 2016a). [The attractor model of decision-making is a neuronal network with associatively modifiable recurrent collateral synapses between the neurons of the type prototypical of the cerebral cortex. The decision variables are applied simultaneously, and the network, after previous training with these decision variables, reaches a state where the population of neurons representing one of the decision variables has a high firing rate (Rolls and Deco 2010; Deco et al. 2013; Rolls 2016a).] The ventromedial prefrontal cortex receives inputs from the orbitofrontal cortex (and also from the ACC).

Value representations in the pregenual cingulate cortex are confirmed by recording studies in monkeys (Rolls 2008; Kolling et al. 2016). For example, Gabbott, Verhagen, Kadohisa and Rolls found neurons in the pregenual cingulate cortex that respond to taste, and it was demonstrated that the representation is of reward value, for devaluation by feeding to satiety selectively decreased neuronal responses to the food with which the animal was satiated (Rolls 2008).

This evidence supports the framework that the value representations computed in the orbitofrontal cortex where there is little representation of action are transferred to the anterior cingulate cortex, where they can be used as the representation of reward vs non-reward or punishment outcome to be associated with representations of actions as part of goal-dependent action–outcome learning.

#### Anterior cingulate cortex and action–outcome representations

Some single neuron studies indicating encoding of actions and outcomes have often involved rather dorsal recordings above the pregenual cingulate cortex in the dorsal ACC (dorsal bank of the cingulate sulcus) (Matsumoto et al. 2007; Luk and Wallis 2009; Kolling et al. 2016). In a similar area, action–outcome associations are represented, in that while a monkey was looking at a visual cue, before an action was made, the activity of ACC neurons was related to the expectation of reward or non-reward (25%), the intention to move or not (25%), or a combination of both reward expectation and intention to move (11%) (Matsumoto et al. 2007). Luk and Wallis (2013) described recordings in the same dorsal ACC area that reflected the outcomes when monkeys made a choice of a left or right lever response to obtain a reward outcome, and also described a weak dissociation for more stimulus–outcome neurons in the orbitofrontal cortex, that is when monkeys had to choose the reward outcome based on which visual stimulus was shown. In the same dorsal anterior cingulate area, neurons were more likely to take into account the costs of the actions needed to obtain rewards, as well as the probability of obtaining the reward, than were orbitofrontal cortex neurons (Kennerley and Wallis 2009; Kennerley et al. 2011; Kolling et al. 2016). In the dorsal ACC, neurons may reflect evidence about the several most recent rewards, and use this to help guide choices (Kolling et al. 2016). More ventrally in the ACC, neurons are more likely to reflect reward outcome rather than primarily actions, and the outcome representation trailed that in the orbitofrontal cortex (Cai and Padoa-Schioppa 2012). These findings are consistent with the hypothesis developed here and elsewhere (Rolls 2019a) that the orbitofrontal cortex represents value but not actions, and takes decisions based on reward value, and that value information is transmitted from the orbitofrontal cortex to the ACC, where there are action-related neurons, and where action–outcome learning takes place.

Foraging studies also implicate the ACC in representing value, and in taking into account costs. For example, some neurons responded at higher rates when the monkeys were about to move to another foraging patch, and the threshold amount of this firing before the monkey switched to a new patch depended on the cost of switching (the delay before foraging in the new patch could resume) (Hayden et al. 2011). Consistent with this, the costs of actions can influence reward value representations in the macaque orbitofrontal cortex, even though the actions themselves are not represented in the orbitofrontal cortex (Cai and Padoa-Schioppa 2019).

In a neuroimaging study that provides evidence that the ACC is active when outcome information guides choices made by the individual (Walton et al. 2004), the activations were relatively far back in the ACC (*y* = 22) towards the midcingulate cortex. This supports the hypothesis provided above that the reward value information in the pregenual cingulate cortex and the negative value representations in the supracallosal ACC are projected posteriorly towards the midcingulate area for interfacing to action.

### Anterior cingulate cortex lesion effects

Lesion studies in humans (Camille et al. 2011) and macaques (Rudebeck et al. 2008) have provided evidence for a dissociation in the role of the ACC in action–outcome associations to guide behaviour; and of the orbitofrontal cortex in stimulus–outcome associations to update the expected value (Rushworth et al. 2012). Lesions of the ACC in rats impair the ability to take account of the costs of actions, and there is a complementary human neuroimaging study (Croxson et al. 2009).

An investigation more closely related to the understanding of emotion showed that patients with selective surgical lesions of the anterior ventral part of the ACC and/or medial prefrontal cortex area BA9 were impaired on voice and face expression identification, had changed social behaviour, such as inappropriateness, and had changes in their subjectively experienced emotions (Hornak et al. 2003). In line with these results in humans, reduced social vocalization and social and emotional changes were found in monkeys with anterior cingulate lesions (Hadland et al. 2003).

Complementing the human anterior cingulate damage effects on voice and in some cases face expression identification (Hornak et al. 2003), neuroimaging studies of vocal expression identification have reported orbital and medial prefrontal/cingulate activation. For example, non-verbal sounds expressing fear, sadness, and happiness activated orbital cortex BA11 and medial BA9 (Morris et al. 1996). Further, fearful sounds activated the ACC (BA32 and BA24) (Phillips et al. 1998). Facial expression identification produces activation in orbital and medial regions that include BA32/24 (anterior cingulate) and medial BA9 (Blair et al. 1999; Dolan et al. 1996; Nakamura et al. 1999).

Complementing the human anterior cingulate damage effects on the subjective experience of emotion (Hornak et al. 2003), activations were found in the ventral ACC and medial BA9 during self-generated emotional experience when participants recalled emotions of sadness or happiness (Lane et al. 1997a, b, 1998).

Based on this evidence, the hypothesis is that the ACC receives inputs from the orbitofrontal cortex and amygdala about expected rewards and punishers, and about the outcomes, the rewards and punishers (which include face and voice expression) received, and that these are used as the value representations in action–outcome learning performed in the cingulate cortex.

## Subgenual cingulate cortex

The subgenual part of the anterior cingulate cortex (areas 25, s24, s32, and the ventral portion of area 33 (Palomero-Gallagher et al. 2015) see Fig. S1) has outputs to the hypothalamus and brainstem autonomic regions, and is involved in the autonomic components of emotion (Koski and Paus 2000; Barbas and Pandya 1989; Ongür and Price 2000; Gabbott et al. 2003; Vogt 2009). Area s32 was co-activated with areas of the executive control network, and was associated with tasks probing cognition in which stimuli did not have an emotional component (Palomero-Gallagher et al. 2015). Area 33 was activated by painful stimuli and co-activated with areas of the sensorimotor network (Palomero-Gallagher et al. 2015). The dorsal anterior and midcingulate cortical areas may be especially related to blood pressure, pupil size, heart rate, and electrodermal activity, whereas the subgenual cingulate cortex, with ventromedial prefrontal cortex, appears antisympathetic (and parasympathetic) (Critchley and Harrison 2013; Nagai et al. 2004). The subgenual cingulate cortex is connected with the ventromedial prefrontal cortical areas (Johansen-Berg et al. 2008).

Evidence implicating the subgenual and more generally the subcallosal cingulate cortex in depression is described elsewhere (Hamani et al. 2011; Laxton et al. 2013; Rolls 2018a; Price and Drevets 2012; Drevets et al. 2008; Holtzheimer et al. 2017). Interestingly, neurons in the human subcallosal cingulate cortex responded to emotion categories present in visual stimuli, with more neurons responding to negatively valenced than positively valenced emotion categories (Laxton et al. 2013).

## Midcingulate cortex, the cingulate motor area, and action–outcome learning

A midcingulate area has been termed the cingulate motor area (Vogt 2009, 2016; Vogt et al. 1996, 2003). The midcingulate area can be divided into an anterior or rostral cingulate motor area (that includes 24c′ and 24a′) involved in skeletomotor control in for example avoidance and fear tasks and in action–outcome learning, and a posterior or caudal cingulate motor area (that includes 24d and p24′) involved in skeletomotor orientation (Vogt et al. 2003; Vogt 2016) (see Fig. S1). [As has been noted above, what has been termed the anterior midcingulate cortex (Vogt 2016) may overlap with or be similar to what is described as the supracallosal part of the ACC in “Anterior cingulate cortex: functional neuroimaging and neuronal activity”.]

This midcingulate cortex is activated by pain, but this may be related to the responses being selected (Derbyshire et al. 1998; Vogt et al. 1996). Social pain, for example being excluded from a social group, can also activate both the anterior cingulate and the midcingulate areas (Eisenberger and Lieberman 2004).

In macaques, lesions of midcingulate and anterior cingulate areas may affect task switching, perhaps because of a difficulty in monitoring error, but do not affect working memory (measured by delayed alternation) (Rushworth et al. 2003, 2004).

The anterior/midcingulate cortex is activated in humans when there is conflict between possible responses, or when there is a change in response set, but not when only stimulus selection is involved (Rushworth et al. 2002).

Errors made in many tasks activate the anterior/midcingulate cortex, whereas tasks with response conflict activate the superior frontal gyrus (Rushworth et al. 2004; Kolling et al. 2016; Procyk et al. 2016), and some anterior/midcingulate neurons respond when errors are made (Niki and Watanabe 1979; Kolling et al. 2016; Procyk et al. 2016), or when rewards are reduced (Shima and Tanji 1998). These errors include errors made for incorrect actions, that is, to action–outcome errors, whereas the error neurons in the orbitofrontal cortex respond to stimulus–reward errors, when the reward is less than expected given the stimulus. Thus, part of the difference is that the orbitofrontal cortex specializes in errors relating to stimuli and rewards associated with them, whereas the cingulate cortex specializes in errors relating to actions and the rewards associated with them (Thorpe et al. 1983; Rolls 2018a, 2019b). The error-related negativity (ERN) potential in humans may originate in area 24c’ (Ullsperger and von Cramon 2001). (ERN is an electroencephalogram negative potential which peaks approximately 50–150 ms after an error is committed by a participant.)

Correspondingly, in rodents a part of the medial prefrontal/anterior cingulate cortex termed the prelimbic cortex is involved in learning relations between actions and reinforcers/outcomes (Balleine and Dickinson 1998; Cardinal et al. 2002; Killcross and Coutureau 2003). The rodent prelimbic cortex may also take into account costs of actions, in that rats with prelimbic cortex lesions were impaired when they had to decide about an action with a large reward but a high barrier to climb, vs an action with a low reward but no barrier (Walton et al. 2002, 2003).

The concept is advanced that the midcingulate cortex may be part of a cingulate system that enables reward outcome information from the orbitofrontal cortex to be associated with action information from the posterior cingulate cortex, with the output directed to premotor cortical areas. Consistent with this, the anterior part of the midcingulate cortex, aMCC in Fig. S1, is described as anticipating and signalling motivationally relevant targets, encoding reward values, signalling errors, and influencing motor responses (Bush 2011). It has been suggested that the aMCC goal and feedback information is used to modulate activity in executive brain regions that direct attention and produce motor responses, and is thereby relevant to attention-deficit hyperactivity disorder (Bush 2011).

## The posterior cingulate cortex

The posterior cingulate cortex receives major inputs from parietal cortical areas that receive from the dorsal visual stream and somatosensory areas, and is involved in spatial processing, action in space, and some types of memory (Vogt 2009; Vogt and Pandya 1987; Vogt and Laureys 2009; Rolls and Wirth 2018; Rolls 2018b). Interestingly, the posterior cingulate cortex also receives connections from the orbitofrontal cortex (Vogt and Pandya 1987; Vogt and Laureys 2009) (Fig. 1). The posterior cingulate cortex is a region with strong connections in primates to the parahippocampal gyrus (areas TF and TH) and the entorhinal cortex, and thus via a dorsal route with the hippocampal memory system (Vogt and Laureys 2009; Rolls and Wirth 2018; Rolls 2018b; Bubb et al. 2017) (Fig. 1). Backprojections from the hippocampal system to posterior cingulate and parietal areas are likely to be involved in memory recall (Kesner and Rolls 2015; Rolls 2016a, 2018b).

One key and interesting concept that emerges is that orbitofrontal cortex value-related information has access to the posterior cingulate cortex and by this dorsal route into the hippocampal memory system, as well as by the ventral route via the perirhinal and (lateral) entorhinal cortex via which object-related information reaches the hippocampal memory system (Fig. 1) (Rolls and Wirth 2018; Rolls 2018b). The hippocampal memory system can then associate these three types of information, about what object or face is present, where it is in space ‘out there’ using spatial view cells, and combining this with information about the reward value of the object or position in space (Rolls 2016a, 2018b; Kesner and Rolls 2015; Rolls and Wirth 2018; Rolls et al. 1997, 1998, 2005; Rolls and Xiang 2005, 2006; Robertson et al. 1998; Georges-François et al. 1999).

Consistent with its anatomy, the posterior cingulate region (BA 23/31) (with the retrosplenial cortex BA 29/30) is consistently engaged by a range of tasks that examine episodic memory including autobiographical memory and imagining the future, and also spatial navigation and scene processing (Leech and Sharp 2014; Auger and Maguire 2013). Self-reflection and self-imagery activate the ventral part of the posterior cingulate cortex (Kircher et al. 2000, 2002; Johnson et al. 2002; Sugiura et al. 2005). I suggest that these memory-related functions, quite understandable in view of the connectivity of the posterior cingulate cortex shown in Fig. 1, account for why it is activated in the resting state and is included as part of the ‘default-mode’ network (Andrews-Hanna et al. 2010; Leech and Sharp 2014).

A second key and interesting concept is that the posterior cingulate cortex and the retrosplenial cortex, which are both highly connected with both lateral and medial parietal cortex areas (Vogt 2009; Vogt and Pandya 1987; Vogt and Laureys 2009; Rolls and Wirth 2018; Rolls 2018b), provide a route for information about actions in space, represented in the parietal cortex by both visual spatial and somatosensory representations (Bisley and Goldberg 2010; Andersen et al. 2000; Andersen 1995; Gnadt and Andersen 1988; Whitlock 2017), to gain access to the cingulate cortex action–outcome learning system. The resulting concept is that the cingulate cortex receives action information via the parietal to posterior cingulate cortex route, and reward information via the orbitofrontal cortex to anterior cingulate cortex route, and from this information associates actions with outcomes, can then select optimal actions given the rewards and costs, and can produce goal-directed actions via the cingulate motor area with its outputs to premotor cortical areas, as illustrated in Fig. 1.

Also consistent with the major anatomical connections of the posterior parietal cortex with both the medial and the lateral parietal cortical areas (Vogt 2009; Vogt and Pandya 1987; Vogt and Laureys 2009), which are involved in spatial function and spatial attention, the posterior parietal cortex has been implicated in spatial attention (Small et al. 2003; Mohanty et al. 2008) and reward-related saccades (McCoy et al. 2003).

The posterior cingulate cortex is implicated in some types of decision-making in that some neurons there respond when risky, uncertain choices are made (McCoy and Platt 2005); and some neurons respond more when an expected large reward is not obtained, maintaining that firing until the next trial (Hayden et al. 2008) [probably reflecting input from orbitofrontal cortex error neurons that have attractor state-like persistent firing that encodes and maintains a negative reward prediction error signal (Thorpe et al. 1983; Rolls and Grabenhorst 2008; Rolls 2019b)].

## The cingulate cortex and depression

Evidence implicating the subgenual and more generally the subcallosal cingulate cortex in depression includes evidence that neurons in this region in humans can respond to unpleasant stimuli; that the subgenual cingulate cortex may be overactive in depression; and that deep brain stimulation may help to treat depression in some individuals (Hamani et al. 2011; Laxton et al. 2013; Rolls 2018a).

In terms of functional connectivity, voxels in the anterior cingulate cortex have higher functional connectivity in unmedicated depressed patients with a number of brain areas (Rolls et al. 2018, 2019b). Higher functional connectivity in depression is found of the subcallosal anterior cingulate with the lateral orbitofrontal cortex; of the pregenual/supracallosal anterior cingulate with the medial orbitofrontal cortex; and of parts of the anterior cingulate with the inferior frontal gyrus. The high functional connectivity in depression between the lateral orbitofrontal cortex and the subcallosal anterior cingulate may relate to more non-reward information transmission to the anterior cingulate, contributing to depression. The high functional connectivity between the medial orbitofrontal cortex and supracallosal anterior cingulate in depression may also contribute to depressive symptoms, in that medial orbitofrontal cortex signals are being routed through a non-reward part of the ACC (Rolls et al. 2018).

In a resting state functional connectivity neuroimaging study of depression, voxels in the posterior cingulate cortex had higher connectivity with the lateral orbitofrontal cortex (Cheng et al. 2018a), involved in non-reward and thereby implicated in depression (Rolls 2016b, 2018a; Rolls et al. 2019b). This connectivity was lower in medicated individuals. It was found in healthy controls that the posterior cingulate cortex has high functional connectivity with the parahippocampal regions which are involved in memory. These discoveries (Cheng et al. 2018a) support the theory that the non-reward system in the lateral orbitofrontal cortex has increased effects on memory systems in depression, which contribute to the rumination about sad memories and events (Rolls 2016b, 2018a).

## Synthesis

### The anterior cingulate cortex and emotion

The anterior cingulate cortex receives information from its topologically nearby neocortical area, the orbitofrontal cortex (most of which is neocortical in primates, and which receives from ventral stream ‘what’ areas), and also the amygdala (also a recipient of ventral stream projections), and projects this information to a number of areas, including autonomic areas in the brainstem as well as in the insula, to the midcingulate cortex, and to the striatum. Because reward value is important in producing emotions, the anterior cingulate cortex becomes involved in emotion (Rolls 2014a, 2018a, 2019a), and damage to it in humans can impair emotions (Hornak et al. 2003). This provides for an emotion-related function of the cingulate cortex.

### Action–outcome learning

Another important function of the cingulate cortex is, via the midcingulate premotor area with its connections to neocortical motor areas, to associate actions with outcomes, as indicated by the connections shown in green in Fig. 1. My proposal is that convergence of reward or outcome information from the ACC, and of information about actions from the posterior cingulate cortex, occurs in the cingulate cortex leading to outputs via the midcingulate motor area, which projects to premotor areas including the premotor cortex area 6 and the supplementary motor area (see green arrows in Fig. 1). This provides connectivity for action–outcome learning (Rolls 2019a). The ACC receives reward and punishment outcome information from the orbitofrontal cortex (OFC). The posterior cingulate cortex receives information about actions from the parietal cortex. Then these two types of information are brought together towards the mid-part of the cingulate cortex including the cingulate premotor area (Vogt 2016), which with its connections to premotor neocortical areas can select the action that is most likely, given the action–outcome learning performed within this cingulate system, to obtain the goal, the desired outcome (Rolls 2019a).

In addition, the parietal areas have projections to medial frontal areas connected with the dorsal parts of the ACC (Vogt 2009), and these projections may also provide a route for action-related information to reach the cingulate action–outcome learning system (Rolls 2019a).

### Connectivity of the cingulate cortex with the hippocampal memory system

A third important function of the cingulate cortex is related to the hippocampal memory system, as shown in Fig. 1. This function is introduced by some anatomical considerations.

The cingulate cortex is largely agranular, that is, it does not have a well-developed layer 4 with granule cells (although a layer 4 is present in areas 23, 31, and 32). However, the cingulate cortex is similar to the neocortex (or isocortex) in other ways, with clear layers 2 and 3, and 5 and 6. Indeed, the cingulate cortex has a high cell density in layers 5 and 6. A term used to describe this cortex is proisocortex (Pandya et al. 2015). Another region of proisocortex is the posterior-most part of the primate orbitofrontal cortex which is agranular and includes the most posterior part of area 13. [The more anterior part of area 13 is granular (Rolls 2019a, b).] Other regions of proisocortex include the agranular anterior insula, the temporal pole, the parahippocampal cortex, and the rostral perirhinal cortex (Pandya et al. 2015). It has been hypothesized that neocortical areas develop in evolution from these proisocortical areas (Pandya et al. 2015).

The connections of these proisocortical areas include forming connectional bridges between neocortical areas and areas such as the hippocampus (which is termed allocortex). Indeed, this is evident in Fig. 1, which shows how perirhinal cortex forms a bridge from the ventral stream neocortical areas (including temporal lobe cortical areas) to the hippocampus (and back), and how the posterior cingulate cortex and parahippocampal cortex form a bridge from the dorsal stream neocortical areas (including the parietal cortex) to the hippocampus (and back) (via the entorhinal cortex in both cases). It is notable that the orbitofrontal cortex and ACC project information, probably about rewards, via both the ventral and dorsal routes, to the hippocampal system (via the perirhinal cortex in the ventral route; and via the posterior cingulate cortex in the dorsal stream to parahippocampal cortex connections).

This is consistent with the framework I propose that the cingulate cortex provides a bridge linking neocortical areas with the hippocampal memory system (Rolls 2018b; Rolls and Wirth 2018), with spatial information reaching the hippocampal system via the posterior cingulate cortex, and reward-related information reaching the hippocampus via the anterior cingulate and orbitofrontal cortex (Rolls 2019a).

This cingulate connectivity is further compared with that of the hippocampus, which receives information from the ventral ‘what’ processing stream (blue) and the dorsal ‘where’ or ‘action’ processing stream (red) in Fig. 1. The entorhinal cortex area 28 is the main entry for cortical connections to and from the hippocampus. The forward projections to the hippocampus are shown with large arrowheads, and the backprojections with small arrowheads. The main ventral stream connections to the hippocampus which convey information about objects, faces, etc. are in blue, and the main dorsal stream connections which convey ‘where’ information about space and movements are in red. The ventral ‘what’ visual pathways project from the primary visual cortex V1 toV2, then V4, then posterior inferior temporal visual cortex (PIT), then anterior inferior temporal visual cortex (AIT), then perirhinal cortex (areas 35/36), and thus to entorhinal cortex (Rolls 2016a). The dorsal ‘where’ visual pathways project from V1 to V2, then MT (middle temporal), then LIP (lateral intraparietal), then parietal area 7 (lateral) and medial (including the precuneus), then to posterior cingulate cortex areas 23/32) including the retrosplenial cortex (areas 29/30) and thus to the parahippocampal gyrus (areas TF and TH), and then entorhinal cortex. Area 22 is the superior temporal auditory association cortex. Reward information reaches the hippocampus from the orbitofrontal cortex (OFC), ACC (areas 32 and 25), and amygdala; but also via the orbitofrontal cortex to posterior cingulate cortex connections. The lateral prefrontal cortex areas 9 and 46 involved in working memory connect via the posterior cingulate cortex. The hippocampus enables all the high-order cortical regions to converge into a single network in the hippocampal CA3 region, which is involved in episodic memory (Rolls 2015, 2016a; Kesner and Rolls 2015).

A difference of hippocampal connectivity therefore from that of the cingulate cortex is that the inputs to the hippocampus from the ventral stream (blue in Fig. 1) are about objects and faces, etc. (from the temporal cortical visual areas), as well as reward information. This fits with the concept that the hippocampus is able to associate together ‘what’ or reward information (blue in Fig. 1) with spatial or temporal information (red in Fig. 1) to enable the formation of episodic memories about objects and faces, and where they were seen on a particular occasion (Rolls 2016a, 2018b; Rolls and Wirth 2018; Kesner and Rolls 2015; Rolls and Kesner 2006). In contrast, it is argued that the cingulate cortex performs the different computational function of associating actions with outcomes, so needs reward and spatial/action information, but less object information, and performs computations that may be based on several recent occasions in which outcomes resulted from actions (Rolls 2019a).

It should be noted that the anatomical connectivity shown in Fig. 1 provides two routes for reward-related information to reach the hippocampal memory system. The first, more direct, route is from the orbitofrontal and amygdala to the perirhinal cortex with information derived from the ventral visual and auditory ‘what’ processing streams (blue in Fig. 1). The second route for reward-related information is via the ACC (which receives its reward-related information from the orbitofrontal cortex) which projects to the posterior cingulate cortex and also to the parahippocampal gyrus, providing access to the hippocampal memory system via the dorsal route. There are also direct projections from the orbitofrontal cortex to the posterior cingulate cortex (green in Fig. 1). The presence of reward-related information in the posterior cingulate cortex and its connected parietal areas is likely to relate to the roles of these areas in some types of decision-making (Hayden et al. 2008; Platt and Glimcher 1999).

## Conclusions

Overall, the conceptualization provided here of the cingulate cortex, a key brain region with isocortical structure, shows how its connections are related to both the ventral and the dorsal cortical processing streams; how its functions are important in emotion, episodic memory, and action–outcome learning; and how its functions are related to those of other limbic structures including the hippocampus, and to the functions of different neocortical areas. Some of the key points that are made, some of which have not been made or emphasized in previous research, are as follows, and relate to what is shown in Fig. 1.

1. The cingulate cortex has several different functions, which can be related to the connections of its different parts. As an area of proisocortex it forms a connectional bridge between neocortical areas and areas such as the hippocampus (which is termed allocortex) (Pandya et al. 2015). The connections can in this way be related to its evolutionary history as a proisocortical structure, and the topological position of its different parts.
2. In this framework, the posterior part, the posterior cingulate cortex, receives information from the nearby neocortical areas such as the parietal cortex about spatial representations, and projects this onwards via the parahippocampal cortex to the hippocampus (which is allocortex), where it provides the spatial component for object–space episodic memories. This provides for a memory-related function of the cingulate cortex.
3. In the same framework, the anterior cingulate cortex receives information from its topologically nearby neocortical area, the orbitofrontal cortex (most of which is neocortical in primates, and which receives from ventral stream ‘what’ areas), and also the amygdala (also a recipient of ventral stream projections), and projects this information to a number of areas, including autonomic areas in the brainstem as well as in the insula, and via midcingulate cortex to premotor cortical areas, and to the striatum. Because reward value is important in producing emotions, the anterior cingulate cortex becomes involved in emotion (Rolls 2014a, 2018a, 2019a). This provides for an emotion-related function of the cingulate cortex.
4. In the same overall framework, the midcingulate cortex has connections to its topologically nearby neocortical areas, which include premotor areas. It is argued here that this provides for a third important function of the cingulate cortex, action–outcome learning, that is, the learning of what instrumental actions to perform to obtain a reward or goal. The cingulate cortex, it turns out, is well connected to perform this third function, because it receives information about the action performed from the parietal areas via the posterior cingulate cortex, and about the reward value of the outcome from the orbitofrontal cortex via the anterior cingulate cortex. These two sources of input can then be related to each other via the connections in the cingulum bundle, and after learning, can then be projected via the midcingulate area to neocortical premotor areas.

The further conclusions add details to the framework just described.

5. The orbitofrontal cortex, which represents value and not actions, projects reward value information to the anterior cingulate cortex. Information about rewards in the medial orbitofrontal cortex reaches the pregenual cingulate cortex, and information about non-reward and punishers represented in the lateral orbitofrontal cortex reaches the supracallosal anterior cingulate cortex. This is shown by activation and functional connectivity studies in humans (Rolls 2018a, 2019a, b).
6. The anterior cingulate cortex represents reward value (received from the orbitofrontal cortex), but also includes representations of actions.
7. A major source of information about actions, it is argued, reaches the cingulate cortex from the parietal cortex, which has major connections with the posterior cingulate cortex (Vogt 2009). The parietal cortex represents actions in terms of body and eye movements in space (lateral parietal cortex), and of the self in a spatial context [medial parietal cortex including the precuneus (Cheng et al. 2018b)].
8. The information from the posterior parietal cortex about actions that are being made is projected towards the middle cingulate cortex (and probably anterior cingulate cortex), where it is associated with reward outcomes in action–outcome learning. The midcingulate cortex has major outputs to premotor areas, to implement the appropriate action given the outcomes received previously for each action. The connections for this are made evident in Fig. 1.
9. The connectivity shown in Fig. 1 shows how the cingulate cortex is related to the hippocampal memory system:

The ventral visual pathways for object identity and the orbitofrontal cortex reward value system project object and reward information via the perirhinal cortex and lateral entorhinal cortex into the hippocampal memory system.

Interestingly, the anterior cingulate cortex projects to the parahippocampal gyrus and via the medial entorhinal cortex into the hippocampus, perhaps taking this route because the ACC represents actions.

The posterior cingulate cortex receives spatial information from the parietal cortex, and projects this spatial information via the parahippocampal gyrus (areas TF and TH) and medial entorhinal cortex into the hippocampal memory system.

The hippocampus can then combine this object, reward, and spatial information in its CA3 network to store episodic memories, as described in detail elsewhere (Rolls 2016a, 2018b; Kesner and Rolls 2015).

However, the information needs to be retrieved from the hippocampus. This utilizes backprojections originating in hippocampal CA1 back to neocortex, through the whole series of backprojections involving the lateral entorhinal cortex, and perirhinal cortex to the ventral stream areas, and orbitofrontal cortex, for the ventral route shown in blue in Fig. 1; and involving the medial entorhinal cortex, parahippocampal gyrus, and posterior cingulate cortex, to the parietal cortex areas, for the dorsal route shown in red in Fig. 1.

This dual backprojection system enables the object information from an episodic memory to be recalled to the ventral stream areas, the reward information to the orbitofrontal cortex, and the spatial information to the parietal cortex (Rolls 2016a, 2018b; Kesner and Rolls 2015).

10. These three functions related to different connectivity of the different parts of the cingulate cortex provide further evidence that we should no longer think of a single limbic system, but instead of multiple limbic systems (Rolls 2015).

One limbic system may involve the amygdala, orbitofrontal cortex, and anterior cingulate cortex for emotion.

A second limbic system may involve the hippocampus, perirhinal and parahippocampal cortex, and posterior cingulate cortex for episodic memory, including object–spatial associations, object and spatial associations with temporal order. Reward information can also enter this hippocampal episodic memory system from the orbitofrontal cortex so that reward value information can be included in an episodic memory (Rolls 2016a, 2018b; Kesner and Rolls 2015; Rolls and Wirth 2018).

A third limbic system may involve associations with the cingulate cortex for action–outcome learning, with the action information entering via the posterior cingulate cortex, the reward value outcome information via the anterior cingulate cortex, and the resulting association directed from the midcingulate cortex to premotor cortical areas.

The cingulate cortex is of interest within this context, as a limbic structure that is involved in all three of these functions, and this is related in this paper to the different connectivity of the different parts of this proisocortical structure.

## Electronic supplementary material

Below is the link to the electronic supplementary material.

Supplementary material 1 (DOCX 256 kb)

## References

[CR1] Andersen RA, Gazzaniga MS (1995). Coordinate transformations and motor planning in posterior parietal cortex. The cognitive neurosciences.

[CR2] Andersen RA, Batista AP, Snyder LH, Buneo CA, Cohen YE, Gazzaniga MS (2000). Programming to look and reach in the posterior parietal cortex. The new cognitive neurosciences.

[CR3] Andrews-Hanna JR, Reidler JS, Sepulcre J, Poulin R, Buckner RL (2010). Functional-anatomic fractionation of the brain’s default network. Neuron.

[CR4] Auger SD, Maguire EA (2013). Assessing the mechanism of response in the retrosplenial cortex of good and poor navigators. Cortex.

[CR5] Balleine BW, Dickinson A (1998). The role of incentive learning in instrumental outcome revaluation by sensory-specific satiety. Anim Learn Behav.

[CR6] Barbas H, Pandya DN (1989). Architecture and intrinsic connections of the prefrontal cortex in the rhesus monkey. J Comp Neurol.

[CR7] Bisley JW, Goldberg ME (2010). Attention, intention, and priority in the parietal lobe. Annu Rev Neurosci.

[CR8] Blair RJ, Morris JS, Frith CD, Perrett DI, Dolan RJ (1999). Dissociable neural responses to facial expressions of sadness and anger. Brain.

[CR9] Broca P (1878). Anatomie comparée des circonvolutions cérébrales: le grand lobe limbique et la scissure limbique dans la série des mammifères. Revue Anthropologique.

[CR10] Bubb EJ, Kinnavane L, Aggleton JP (2017). Hippocampal—diencephalic—cingulate networks for memory and emotion: An anatomical guide. Brain Neurosci Adv.

[CR11] Bush G (2011). Cingulate, frontal, and parietal cortical dysfunction in attention-deficit/hyperactivity disorder. Biol Psychiatry.

[CR12] Cai X, Padoa-Schioppa C (2012). Neuronal encoding of subjective value in dorsal and ventral anterior cingulate cortex. J Neurosci.

[CR13] Cai X, Padoa-Schioppa C (2019). Neuronal evidence for good-based economic decisions under variable action costs. Nat Commun.

[CR14] Camille N, Tsuchida A, Fellows LK (2011). Double dissociation of stimulus-value and action-value learning in humans with orbitofrontal or anterior cingulate cortex damage. J Neurosci.

[CR15] Cardinal N, Parkinson JA, Hall J, Everitt BJ (2002). Emotion and motivation: the role of the amygdala, ventral striatum, and prefrontal cortex. Neurosci Biobehav Rev.

[CR16] Carmichael ST, Price JL (1995). Limbic connections of the orbital and medial prefrontal cortex in macaque monkeys. J Comp Neurol.

[CR17] Carmichael ST, Price JL (1996). Connectional networks within the orbital and medial prefrontal cortex of macaque monkeys. J Comp Neurol.

[CR18] Cavanna AE, Trimble MR (2006). The precuneus: a review of its functional anatomy and behavioural correlates. Brain.

[CR19] Cheng W, Rolls ET, Qiu J, Xie X, Wei D, Huang CC, Yang AC, Tsai SJ, Li Q, Meng J, Lin CP, Xie P, Feng J (2018). Increased functional connectivity of the posterior cingulate cortex with the lateral orbitofrontal cortex in depression. Transl Psychiatry.

[CR20] Cheng W, Rolls ET, Qiu J, Yang D, Ruan H, Wei D, Zhao L, Meng J, Xie P, Feng J (2018). Functional connectivity of the precuneus in unmedicated patients with depression. Biol Psychiatry Cogn Neurosci Neuroimaging.

[CR21] Critchley HD, Harrison NA (2013). Visceral influences on brain and behavior. Neuron.

[CR22] Croxson PL, Walton ME, O’Reilly JX, Behrens TE, Rushworth MF (2009). Effort-based cost-benefit valuation and the human brain. J Neurosci.

[CR23] de Araujo IET, Rolls ET (2004). The representation in the human brain of food texture and oral fat. J Neurosci.

[CR24] de Araujo IET, Kringelbach ML, Rolls ET, Hobden P (2003). The representation of umami taste in the human brain. J Neurophysiol.

[CR25] de Araujo IET, Kringelbach ML, Rolls ET, McGlone F (2003). Human cortical responses to water in the mouth, and the effects of thirst. J Neurophysiol.

[CR26] de Araujo IET, Rolls ET, Velazco MI, Margot C, Cayeux I (2005). Cognitive modulation of olfactory processing. Neuron.

[CR27] Deco G, Rolls ET, Albantakis L, Romo R (2013). Brain mechanisms for perceptual and reward-related decision-making. Prog Neurobiol.

[CR28] Derbyshire SWG, Vogt BA, Jones AKP (1998). Pain and Stroop interference tasks activate separate processing modules in anterior cingulate cortex. Exp Brain Res.

[CR29] Devinsky O, Morrell MJ, Vogt BA (1995). Contributions of anterior cingulate cortex to behaviour. Brain.

[CR30] Dolan RJ, Fletcher P, Morris J, Kapur N, Deakin JF, Frith CD (1996). Neural activation during covert processing of positive emotional facial expressions. Neuroimage.

[CR31] Drevets WC, Savitz J, Trimble M (2008). The subgenual anterior cingulate cortex in mood disorders. CNS Spectr.

[CR32] Eisenberger NI, Lieberman MD (2004). Why rejection hurts: a common neural alarm system for physical and social pain. Trends Cogn Sci.

[CR33] Gabbott PL, Warner TA, Jays PR, Bacon SJ (2003). Areal and synaptic interconnectivity of prelimbic (area 32), infralimbic (area 25) and insular cortices in the rat. Brain Res.

[CR34] Georges-François P, Rolls ET, Robertson RG (1999). Spatial view cells in the primate hippocampus: allocentric view not head direction or eye position or place. Cereb Cortex.

[CR35] Glascher J, Adolphs R, Damasio H, Bechara A, Rudrauf D, Calamia M, Paul LK, Tranel D (2012). Lesion mapping of cognitive control and value-based decision making in the prefrontal cortex. Proc Natl Acad Sci USA.

[CR36] Gnadt JW, Andersen RA (1988). Memory related motor planning activity in posterior parietal cortex of macaque. Exp Brain Res.

[CR37] Grabenhorst F, Rolls ET (2008). Selective attention to affective value alters how the brain processes taste stimuli. Eur J Neurosci.

[CR38] Grabenhorst F, Rolls ET (2011). Value, pleasure, and choice in the ventral prefrontal cortex. Trends Cogn Sci.

[CR39] Grabenhorst F, Rolls ET, Bilderbeck A (2008). How cognition modulates affective responses to taste and flavor: top down influences on the orbitofrontal and pregenual cingulate cortices. Cereb Cortex.

[CR40] Grabenhorst F, Rolls ET, Parris BA (2008). From affective value to decision-making in the prefrontal cortex. Eur J Neurosci.

[CR41] Grabenhorst F, D’Souza A, Parris BA, Rolls ET, Passingham RE (2010). A common neural scale for the subjective pleasantness of different primary rewards. Neuroimage.

[CR42] Grabenhorst F, Rolls ET, Parris BA, D’Souza A (2010). How the brain represents the reward value of fat in the mouth. Cereb Cortex.

[CR43] Grattan LE, Glimcher PW (2014). Absence of spatial tuning in the orbitofrontal cortex. PLoS One.

[CR44] Hadland KA, Rushworth MF, Gaffan D, Passingham RE (2003). The effect of cingulate lesions on social behaviour and emotion. Neuropsychologia.

[CR45] Hamani C, Mayberg H, Stone S, Laxton A, Haber S, Lozano AM (2011). The subcallosal cingulate gyrus in the context of major depression. Biol Psychiatry.

[CR46] Hasselmo ME, Rolls ET, Baylis GC (1989). The role of expression and identity in the face-selective responses of neurons in the temporal visual cortex of the monkey. Behav Brain Res.

[CR47] Hasselmo ME, Rolls ET, Baylis GC, Nalwa V (1989). Object-centred encoding by face-selective neurons in the cortex in the superior temporal sulcus of the monkey. Exp Brain Res.

[CR48] Hayden BY, Nair AC, McCoy AN, Platt ML (2008). Posterior cingulate cortex mediates outcome-contingent allocation of behavior. Neuron.

[CR49] Hayden BY, Pearson JM, Platt ML (2011). Neuronal basis of sequential foraging decisions in a patchy environment. Nat Neurosci.

[CR50] Holtzheimer PE, Husain MM, Lisanby SH, Taylor SF, Whitworth LA, McClintock S, Slavin KV, Berman J, McKhann GM, Patil PG, Rittberg BR, Abosch A, Pandurangi AK, Holloway KL, Lam RW, Honey CR, Neimat JS, Henderson JM, DeBattista C, Rothschild AJ, Pilitsis JG, Espinoza RT, Petrides G, Mogilner AY, Matthews K, Peichel D, Gross RE, Hamani C, Lozano AM, Mayberg HS (2017). Subcallosal cingulate deep brain stimulation for treatment-resistant depression: a multisite, randomised, sham-controlled trial. Lancet Psychiatry.

[CR51] Hornak J, Bramham J, Rolls ET, Morris RG, O’Doherty J, Bullock PR, Polkey CE (2003). Changes in emotion after circumscribed surgical lesions of the orbitofrontal and cingulate cortices. Brain.

[CR52] Insausti R, Amaral DG, Cowan WM (1987). The entorhinal cortex of the monkey. II. Cortical afferents. J Comp Neurol.

[CR53] Izquierdo A (2017). Functional heterogeneity within rat orbitofrontal cortex in reward learning and decision making. J Neurosci.

[CR54] Johansen-Berg H, Gutman DA, Behrens TE, Matthews PM, Rushworth MF, Katz E, Lozano AM, Mayberg HS (2008). Anatomical connectivity of the subgenual cingulate region targeted with deep brain stimulation for treatment-resistant depression. Cereb Cortex.

[CR55] Johnson SC, Baxter LC, Wilder LS, Pipe JG, Heiserman JE, Prigatano GP (2002). Neural correlates of self-reflection. Brain.

[CR56] Kennerley SW, Wallis JD (2009). Evaluating choices by single neurons in the frontal lobe: outcome value encoded across multiple decision variables. Eur J Neurosci.

[CR57] Kennerley SW, Walton ME, Behrens TE, Buckley MJ, Rushworth MF (2006). Optimal decision making and the anterior cingulate cortex. Nat Neurosci.

[CR58] Kennerley SW, Behrens TE, Wallis JD (2011). Double dissociation of value computations in orbitofrontal and anterior cingulate neurons. Nat Neurosci.

[CR59] Kesner RP, Rolls ET (2015). A computational theory of hippocampal function, and tests of the theory: new developments. Neurosci Biobehav Rev.

[CR60] Killcross S, Coutureau E (2003). Coordination of actions and habits in the medial prefrontal cortex of rats. Cereb Cortex.

[CR61] Kircher TT, Senior C, Phillips ML, Benson PJ, Bullmore ET, Brammer M, Simmons A, Williams SC, Bartels M, David AS (2000). Towards a functional neuroanatomy of self processing: effects of faces and words. Brain Res Cogn Brain Res.

[CR62] Kircher TT, Brammer M, Bullmore E, Simmons A, Bartels M, David AS (2002). The neural correlates of intentional and incidental self processing. Neuropsychologia.

[CR63] Kolling N, Wittmann MK, Behrens TE, Boorman ED, Mars RB, Rushworth MF (2016). Value, search, persistence and model updating in anterior cingulate cortex. Nat Neurosci.

[CR64] Koski L, Paus T (2000). Functional connectivity of the anterior cingulate cortex within the human frontal lobe: a brain-mapping meta-analysis. Exp Brain Res.

[CR65] Kringelbach ML, O’Doherty J, Rolls ET, Andrews C (2003). Activation of the human orbitofrontal cortex to a liquid food stimulus is correlated with its subjective pleasantness. Cereb Cortex.

[CR66] Lane RD, Reiman EM, Ahern GL, Schwartz GE, Davidson RJ (1997). Neuroanatomical correlates of happiness, sadness, and disgust. Am J Psychiatry.

[CR67] Lane RD, Reiman EM, Bradley MM, Lang PJ, Ahern GL, Davidson RJ, Schwartz GE (1997). Neuroanatomical correlates of pleasant and unpleasant emotion. Neuropsychologia.

[CR68] Lane RD, Reiman E, Axelrod B, Yun L-S, Holmes AH, Schwartz GE (1998). Neural correlates of levels of emotional awareness. Evidence of an interaction between emotion and attention in the anterior cingulate cortex. J Cogn Neurosci.

[CR69] Laxton AW, Neimat JS, Davis KD, Womelsdorf T, Hutchison WD, Dostrovsky JO, Hamani C, Mayberg HS, Lozano AM (2013). Neuronal coding of implicit emotion categories in the subcallosal cortex in patients with depression. Biol Psychiatry.

[CR70] Leech R, Sharp DJ (2014). The role of the posterior cingulate cortex in cognition and disease. Brain.

[CR71] Luk CH, Wallis JD (2009). Dynamic encoding of responses and outcomes by neurons in medial prefrontal cortex. J Neurosci.

[CR72] Luk CH, Wallis JD (2013). Choice coding in frontal cortex during stimulus-guided or action-guided decision-making. J Neurosci.

[CR73] Matsumoto M, Matsumoto K, Abe H, Tanaka K (2007). Medial prefrontal selectivity signalling prediction errors of action values. Nat Neurosci.

[CR74] McCabe C, Rolls ET, Bilderbeck A, McGlone F (2008). Cognitive influences on the affective representation of touch and the sight of touch in the human brain. Soc Cogn Affect Neurosci.

[CR75] McCoy AN, Platt ML (2005). Risk-sensitive neurons in macaque posterior cingulate cortex. Nat Neurosci.

[CR76] McCoy AN, Crowley JC, Haghighian G, Dean HL, Platt ML (2003). Saccade reward signals in posterior cingulate cortex. Neuron.

[CR77] Mohanty A, Gitelman DR, Small DM, Mesulam MM (2008). The spatial attention network interacts with limbic and monoaminergic systems to modulate motivation-induced attention shifts. Cereb Cortex.

[CR78] Morecraft RJ, Tanji J, Vogt BA (2009). Cingulofrontal interaction and the cingulate motor areas. Cingulate neurobiology and disease.

[CR79] Morris JS, Frith CD, Perrett DI, Rowland D, Young AW, Calder AJ, Dolan RJ (1996). A differential neural response in the human amygdala to fearful and happy facial expressions. Nature.

[CR80] Nagai Y, Critchley HD, Featherstone E, Trimble MR, Dolan RJ (2004). Activity in ventromedial prefrontal cortex covaries with sympathetic skin conductance level: a physiological account of a “default mode” of brain function. Neuroimage.

[CR81] Nakamura K, Kawashima R, Ito K, Sugiura M, Kato T, Nakamura A, Hatano K, Nagumo S, Kubota K, Fukuda H, Kojima S (1999). Activation of the right inferior frontal cortex during assessment of facial emotion. J Neurophysiol.

[CR82] Niki H, Watanabe M (1979). Prefrontal and cingulate unit activity during timing behavior in the monkey. Brain Res.

[CR83] Noonan MP, Mars RB, Rushworth MF (2011). Distinct roles of three frontal cortical areas in reward-guided behavior. J Neurosci.

[CR84] O’Doherty J, Kringelbach ML, Rolls ET, Hornak J, Andrews C (2001). Abstract reward and punishment representations in the human orbitofrontal cortex. Nat Neurosci.

[CR85] Ongür D, Price JL (2000). The organisation of networks within the orbital and medial prefrontal cortex of rats, monkeys and humans. Cereb Cortex.

[CR86] Öngür D, Ferry AT, Price JL (2003). Architectonic division of the human orbital and medial prefrontal cortex. J Comp Neurol.

[CR87] Padoa-Schioppa C, Assad JA (2006). Neurons in the orbitofrontal cortex encode economic value. Nature.

[CR88] Palomero-Gallagher N, Eickhoff SB, Hoffstaedter F, Schleicher A, Mohlberg H, Vogt BA, Amunts K, Zilles K (2015). Functional organization of human subgenual cortical areas: relationship between architectonical segregation and connectional heterogeneity. Neuroimage.

[CR89] Pandya DN, Seltzer B, Petrides M, Cipolloni PB (2015). Cerebral cortex: architecture, connections, and the dual origin concept.

[CR90] Passingham REP, Wise SP (2012). The neurobiology of the prefrontal cortex.

[CR91] Pessoa L, Hof PR (2015). From Paul Broca’s great limbic lobe to the limbic system. J Comp Neurol.

[CR92] Phillips ML, Young AW, Scott SK, Calder AJ, Andrew C, Giampietro V, Williams SC, Bullmore ET, Brammer M, Gray JA (1998). Neural responses to facial and vocal expressions of fear and disgust. Proc R Soc Lond B Biol Sci.

[CR93] Platt ML, Glimcher PW (1999). Neural correlates of decision variables in parietal cortex. Nature.

[CR94] Price JL, Zald DH, Rauch SL (2006). Architectonic structure of the orbital and medial prefrontal cortex. The orbitofrontal cortex.

[CR95] Price JL, Zald DH, Rauch SL (2006). Connections of orbital cortex. The orbitofrontal cortex.

[CR96] Price JL, Drevets WC (2012). Neural circuits underlying the pathophysiology of mood disorders. Trends Cogn Sci.

[CR97] Procyk E, Wilson CR, Stoll FM, Faraut MC, Petrides M, Amiez C (2016). Midcingulate motor map and feedback detection: converging data from humans and monkeys. Cereb Cortex.

[CR98] Rempel-Clower NL, Barbas H (1998). Topographic organization of connections between the hypothalamus and prefrontal cortex in the rhesus monkey. J Comp Neurol.

[CR99] Robertson RG, Rolls ET, Georges-François P (1998). Spatial view cells in the primate hippocampus: effects of removal of view details. J Neurophysiol.

[CR100] Rolls ET (2000). The orbitofrontal cortex and reward. Cereb Cortex.

[CR101] Rolls ET (2008). Functions of the orbitofrontal and pregenual cingulate cortex in taste, olfaction, appetite and emotion. Acta Physiol Hung.

[CR102] Rolls ET, Vogt BA (2009). The anterior and midcingulate cortices and reward. Cingulate neurobiology and disease.

[CR103] Rolls ET (2014). Emotion and decision-making explained.

[CR104] Rolls ET (2014). Emotion and decision-making explained: précis. Cortex.

[CR105] Rolls ET (2015). Limbic systems for emotion and for memory, but no single limbic system. Cortex.

[CR106] Rolls ET (2016). Cerebral cortex: principles of operation.

[CR107] Rolls ET (2016). A non-reward attractor theory of depression. Neurosci Biobehav Rev.

[CR108] Rolls ET (2018). The brain, emotion, and depression.

[CR109] Rolls ET (2018). The storage and recall of memories in the hippocampo-cortical system. Cell Tissue Res.

[CR110] Rolls ET (2019). The orbitofrontal cortex.

[CR111] Rolls ET (2019). The orbitofrontal cortex and emotion in health and disease, including depression. Neuropsychologia.

[CR112] Rolls ET, Deco G (2010). The noisy brain: stochastic dynamics as a principle of brain function.

[CR113] Rolls ET, Grabenhorst F (2008). The orbitofrontal cortex and beyond: from affect to decision-making. Prog Neurobiol.

[CR114] Rolls ET, Kesner RP (2006). A computational theory of hippocampal function, and empirical tests of the theory. Prog Neurobiol.

[CR115] Rolls ET, Wirth S (2018). Spatial representations in the primate hippocampus, and their functions in memory and navigation. Prog Neurobiol.

[CR116] Rolls ET, Xiang J-Z (2005). Reward-spatial view representations and learning in the hippocampus. J Neurosci.

[CR117] Rolls ET, Xiang J-Z (2006). Spatial view cells in the primate hippocampus, and memory recall. Rev Neurosci.

[CR118] Rolls ET, Sienkiewicz ZJ, Yaxley S (1989). Hunger modulates the responses to gustatory stimuli of single neurons in the caudolateral orbitofrontal cortex of the macaque monkey. Eur J Neurosci.

[CR119] Rolls ET, Robertson RG, Georges-François P (1997). Spatial view cells in the primate hippocampus. Eur J Neurosci.

[CR120] Rolls ET, Treves A, Robertson RG, Georges-François P, Panzeri S (1998). Information about spatial view in an ensemble of primate hippocampal cells. J Neurophysiol.

[CR121] Rolls ET, Kringelbach ML, de Araujo IET (2003). Different representations of pleasant and unpleasant odors in the human brain. Eur J Neurosci.

[CR122] Rolls ET, O’Doherty J, Kringelbach ML, Francis S, Bowtell R, McGlone F (2003). Representations of pleasant and painful touch in the human orbitofrontal and cingulate cortices. Cereb Cortex.

[CR123] Rolls ET, Xiang J-Z, Franco L (2005). Object, space and object-space representations in the primate hippocampus. J Neurophysiol.

[CR124] Rolls ET, Grabenhorst F, Margot C, da Silva MAAP, Velazco MI (2008). Selective attention to affective value alters how the brain processes olfactory stimuli. J Cogn Neurosci.

[CR125] Rolls ET, Grabenhorst F, Parris BA (2008). Warm pleasant feelings in the brain. Neuroimage.

[CR126] Rolls ET, McCabe C, Redoute J (2008). Expected value, reward outcome, and temporal difference error representations in a probabilistic decision task. Cereb Cortex.

[CR127] Rolls ET, Grabenhorst F, Deco G (2010). Choice, difficulty, and confidence in the brain. Neuroimage.

[CR128] Rolls ET, Grabenhorst F, Deco G (2010). Decision-making, errors, and confidence in the brain. J Neurophysiol.

[CR129] Rolls ET, Joliot M, Tzourio-Mazoyer N (2015). Implementation of a new parcellation of the orbitofrontal cortex in the automated anatomical labeling atlas. Neuroimage.

[CR130] Rolls ET, Cheng W, Gong W, Qiu J, Zhou C, Zhang J, Lv W, Ruan H, Wei D, Cheng K, Meng J, Xie P, Feng J (2018). Functional connectivity of the anterior cingulate cortex in depression and in health. Cereb Cortex.

[CR131] Rolls ET, Cheng W, Du J, Wei D, Qiu J, Dai D, Zhou Q, Xie P, Feng J (2019a) Functional connectivity of the right inferior frontal gyrus and orbitofrontal cortex in depression **(in review)** 10.1093/scan/nsaa014 PMC7171374 31993660

[CR132] Rolls Edmund T. (2019). The orbitofrontal cortex, depression, and other mental disorders. The Orbitofrontal Cortex.

[CR133] Rudebeck PH, Behrens TE, Kennerley SW, Baxter MG, Buckley MJ, Walton ME, Rushworth MF (2008). Frontal cortex subregions play distinct roles in choices between actions and stimuli. J Neurosci.

[CR134] Rushworth MF, Hadland KA, Paus T, Sipila PK (2002). Role of the human medial frontal cortex in task switching: a combined fMRI and TMS study. J Neurophysiol.

[CR135] Rushworth MF, Hadland KA, Gaffan D, Passingham RE (2003). The effect of cingulate cortex lesions on task switching and working memory. J Cogn Neurosci.

[CR136] Rushworth MF, Walton ME, Kennerley SW, Bannerman DM (2004). Action sets and decisions in the medial frontal cortex. Trends Cogn Sci.

[CR137] Rushworth MF, Buckley MJ, Behrens TE, Walton ME, Bannerman DM (2007). Functional organization of the medial frontal cortex. Curr Opin Neurobiol.

[CR138] Rushworth MF, Noonan MP, Boorman ED, Walton ME, Behrens TE (2011). Frontal cortex and reward-guided learning and decision-making. Neuron.

[CR139] Rushworth MF, Kolling N, Sallet J, Mars RB (2012). Valuation and decision-making in frontal cortex: one or many serial or parallel systems?. Curr Opin Neurobiol.

[CR140] Saleem KS, Kondo H, Price JL (2008). Complementary circuits connecting the orbital and medial prefrontal networks with the temporal, insular, and opercular cortex in the macaque monkey. J Comp Neurol.

[CR141] Shima K, Tanji J (1998). Role for cingulate motor area cells in voluntary movement selection based on reward. Science.

[CR142] Small DM, Zatorre RJ, Dagher A, Evans AC, Jones-Gotman M (2001). Changes in brain activity related to eating chocolate: from pleasure to aversion. Brain.

[CR143] Small DM, Gitelman DR, Gregory MD, Nobre AC, Parrish TB, Mesulam MM (2003). The posterior cingulate and medial prefrontal cortex mediate the anticipatory allocation of spatial attention. Neuroimage.

[CR144] Strait CE, Blanchard TC, Hayden BY (2014). Reward value comparison via mutual inhibition in ventromedial prefrontal cortex. Neuron.

[CR145] Sugiura M, Watanabe J, Maeda Y, Matsue Y, Fukuda H, Kawashima R (2005). Cortical mechanisms of visual self-recognition. Neuroimage.

[CR146] Thorpe SJ, Rolls ET, Maddison S (1983). Neuronal activity in the orbitofrontal cortex of the behaving monkey. Exp Brain Res.

[CR147] Tremblay L, Schultz W (1999). Relative reward preference in primate orbitofrontal cortex. Nature.

[CR148] Ullsperger M, von Cramon DY (2001). Subprocesses of performance monitoring: a dissociation of error processing and response competition revealed by event-related fMRI and ERPs. Neuroimage.

[CR149] Ungerleider LG, Haxby JV (1994). ‘What’ and ‘where’ in the human brain. Curr Opin Neurobiol.

[CR150] Van Essen DC, Hayashi T, Autio J, Ose T, Nishigori K, Coalsor T, Hou Y, Smith S, Shen Z, Knoblauch K, Kennedy H, Glasser M (2019) Evaluation of functional connectivity using retrograde tracers in the macaque monkey. Organisation for Human Brain Mapping. https://ww5.aievolution/hmb1901. Accessed Aug 2019

[CR151] Vogt BA (2009). Cingulate neurobiology and disease.

[CR152] Vogt BA (2016). Midcingulate cortex: structure, connections, homologies, functions and diseases. J Chem Neuroanat.

[CR153] Vogt BA (2019). Handbook of clinical neurology: cingulate cortex. handbook of clinical neurology.

[CR154] Vogt BA, Laureys S, Vogt BA (2009). The primate posterior cingulate gyrus: connections, sensorimotor orientation, gateway to limbic processing. Cingulate neurobiology and disease.

[CR155] Vogt BA, Pandya DN (1987). Cingulate cortex of the rhesus monkey: II. Cortical afferents. J Comp Neurol.

[CR156] Vogt BA, Sikes RW (2000). The medial pain system, cingulate cortex, and parallel processing of nociceptive information. Prog Brain Res.

[CR157] Vogt BA, Derbyshire S, Jones AKP (1996). Pain processing in four regions of human cingulate cortex localized with co-registered PET and MR imaging. Eur J Neurosci.

[CR158] Vogt BA, Berger GR, Derbyshire SW (2003). Structural and functional dichotomy of human midcingulate cortex. Eur J Neurosci.

[CR159] Walton ME, Bannerman DM, Rushworth MF (2002). The role of rat medial frontal cortex in effort-based decision making. J Neurosci.

[CR160] Walton ME, Bannerman DM, Alterescu K, Rushworth MF (2003). Functional specialization within medial frontal cortex of the anterior cingulate for evaluating effort-related decisions. J Neurosci.

[CR161] Walton ME, Devlin JT, Rushworth MF (2004). Interactions between decision making and performance monitoring within prefrontal cortex. Nat Neurosci.

[CR162] Whitlock JR (2017). Posterior parietal cortex. Curr Biol.

[CR163] Wikenheiser AM, Schoenbaum G (2016). Over the river, through the woods: cognitive maps in the hippocampus and orbitofrontal cortex. Nat Rev Neurosci.

