# Supplementary material for: The cingulate cortex and limbic systems for emotion, action, and memory
Source: Brain Struct Funct. 2019 Aug 26;224(9):3001–18. doi: 10.1007/s00429-019-01945-2 (PMC6875144; doi:10.1007/s00429-019-01945-2)
Supplement: Supplementary file 1 — Supplementary material 1 (DOCX 256 kb) [file 429_2019_1945_MOESM1_ESM.docx]

**The Cingulate Cortex and limbic systems for emotion, action, and memory**

**Supplementary Material**

Edmund T. Rolls

Oxford Centre for Computational Neuroscience, Oxford, UK, and

University of Warwick, Department of Computer Science, Coventry, CV4 7AL, UK

**Cytoarchitectural divisions of the human cingulate cortex as described by Vogt**

The cytoarchitectural divisions of the cingulate cortex in humans as described by Vogt (2016, 2009) are shown in Fig. S1 in a flat map. The subgenual cortex is area 25. The anterior cingulate cortex includes the parts of 24 and 32 anterior to the arrow in Fig. S1 showing the anterior part of the midcingulate cortex (aMCC). pMCC is the posterior part of the midcingulate cortex. The posterior cingulate cortex is posterior to the midcingulate cortex and contains areas 23 and area 31. The retrosplenial cortex is areas 29 and 30.


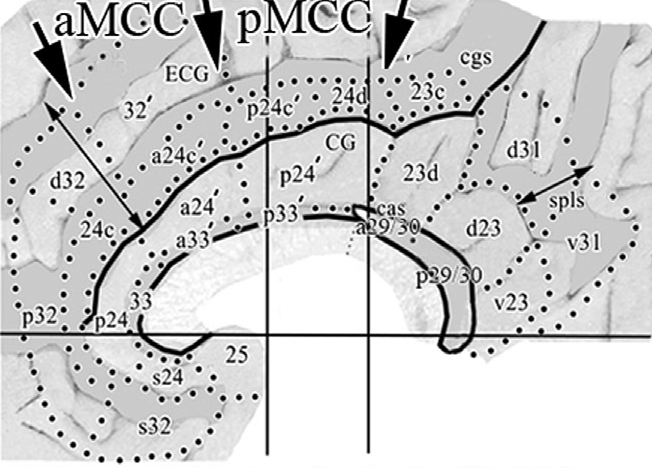


Fig. S1. The cytoarchitectural divisions of the human cingulate cortex as described by Vogt (2016, 2009) shown in a flat map. (Reproduced with permission from Vogt BA (2016) Midcingulate cortex: Structure, connections, homologies, functions and diseases. J Chem Neuroanat 74:28-46.)

References

Vogt BA (ed) (2009) Cingulate Neurobiology and Disease. Oxford University Press, Oxford

Vogt BA (2016) Midcingulate cortex: Structure, connections, homologies, functions and diseases. J Chem Neuroanat 74:28-46. doi:10.1016/j.jchemneu.2016.01.010
